# Locking compression plate distal ulna hook plate fixation versus intramedullary screw fixation for displaced avulsion fifth Metatarsal Base fractures: a comparative retrospective cohort study

**DOI:** 10.1186/s12891-017-1766-z

**Published:** 2017-09-26

**Authors:** Lin Xie, Xin Guo, Shu-Jun Zhang, Zhen-Hua Fang

**Affiliations:** Department of Orthopedic Surgery, Wuhan Orthopedic Hospital, Wuhan Puai Hospital, Huazhong University of Science and Technology, Hanzheng Street 473#, Wuhan City, Hubei Province 430033 China

**Keywords:** Locking compression plate (LCP), Intramedullary screw (IMS), Fifth Metatarsal Base fractures (FMBFs)

## Abstract

**Background:**

Intramedullary screw (IMS) fixation was wildly used in fifth metatarsal base fractures (FMBFs) and the results were satisfactory. However, in the comminuted osteoporosis or small displaced avulsion FMBFs, anatomical reduction and stable fixation could not be achieved with IMS. The Locking Compression Plate (LCP) distal ulna hook plate fixation was a novel alternative fixation method. The aim of this retrospective cohort study was to determine if LCP distal ulna hook plate fixation resulted in improved outcomes compared to the traditional IMS fixation in displaced avulsion FMBFs.

**Methods:**

Of 43 patients with displaced avulsion FMBFs, 18 patients were treated with LCP distal ulna hook plate fixation and 25 were treated with IMS fixation. The patients were evaluated clinically and radiographically and followed up to 12 months. The surgery time, time for hospital stay, time for weight-bearing, time for bony union, time for return to daily life, pain relief, functional outcome and complications after treatment with LCP distal ulna hook plate fixation or IMS fixation were compared. The functional outcome was assessed by the AOFAS (American Orthopedic Foot and Ankle Society) mid-foot score at 3, 6, 9, and 12 months after surgery. Meanwhile, pain scores were obtained at 3, 6, 9, and 12 months after surgery.

**Results:**

The two cohorts had similar baseline characteristics. Surgery time was less in LCP distal ulna hook plate fixation cohort compare to IMS fixation cohort (*p* < 0.0001). Time for partial weight-bearing (*p* < 0.0001) and full weight-bearing (*p* < 0.0001) also demonstrated significant improvements in patients with LCP distal ulna hook plate fixation compared to IMS fixation. Patients in the LCP distal ulna hook plate fixation cohort had significantly increased AOFAS at 9 months (*p* < 0.0001) and 12 months (*p* < 0.0001) after surgery compared to the IMS fixation cohort.

**Conclusion:**

In this retrospective cohort study, LCP distal ulna hook plate fixation as an alternative fixation method was better therapy for the displaced avulsion FMBFs compared to IMS fixation. LCP distal ulna hook plate fixation had a short surgery time and improved functional performance.

**Electronic supplementary material:**

The online version of this article (10.1186/s12891-017-1766-z) contains supplementary material, which is available to authorized users.

## Background

**Fig. 1:**
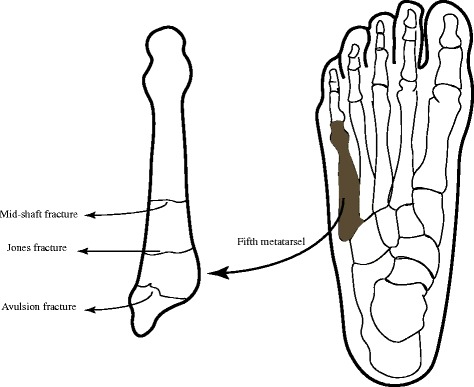
Illustration of classification of fifth metatarsal base fractures

The base of fifth metatarsal was defined as the proximal 1.5 cm of the shaft distal to its articular surface. Fifth metatarsal base fractures (FMBFs) were the most common type of the fifth metatarsal fractures [1]. The fracture typically occurred when an adduction force was applied to the forefoot with the ankle plantarflexed. The base was usually divided anatomically into three zones [2] (its tuberosity, meta-diaphyseal junction and proximal shaft). Fractures in zone 1 were the most familiar ones which comprised about 93% of all the proximal fifth metatarsal fractures [1]. In our study, displaced avulsion FMBFs were defined as the displaced fractures in zone 1 [3, 4] (Fig. 1).

**Fig. 2:**
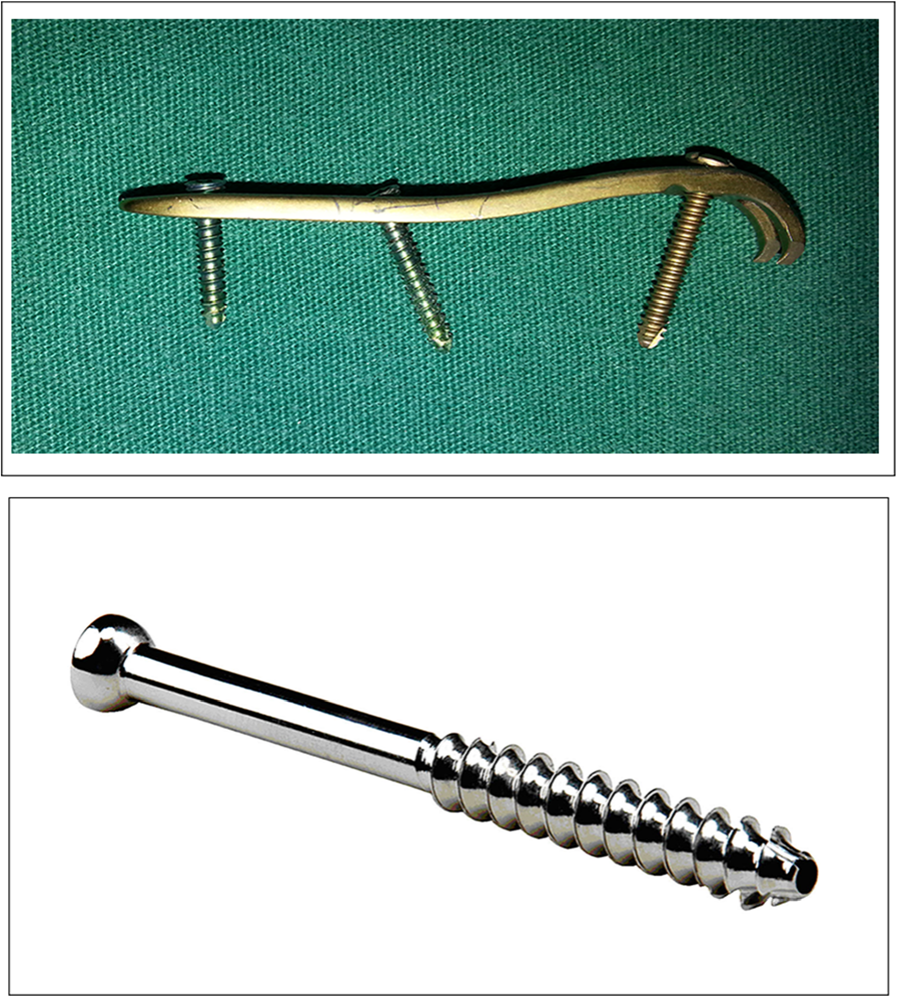
Photograph of Locking Compression Plate (LCP) distal ulna hook plate and Intramedullary Screw

Most un-displaced avulsion FMBFs can be treated with conservative treatment [3, 5]. Non-weight-bearing with cast immobilization has been the main treatment of this injury [5, 6]. However, this injury often was a source of lost work productivity and associated with nonunion rates of 7% to 28% [2]. Fractures displaced more than 2 mm frequently required surgical treatment to achieve anatomic reduction of the articular surface, early weight-bearing and restoration of peroneus longus and brevis tendons [2]. Surgery treatment of displaced avulsion FMBFs was challenging because of its subcutaneous location, comminuted fracture, its attachment of peroneus longus and brevis muscles and its weight-bearing function [3, 7]. Intramedullary screw (IMS) fixation was wildly used in FMBFs [7–9] (Fig. 2). Previous studies showed that the screw was larger in both diameter and length the better [10]. However, the comminuted or small displaced avulsion FMBFs were difficulty to treat with large IMS and function outcomes following IMS remained unsatisfactory because of late weight-bearing [11]. Meanwhile, the IMS was associated with many complications, including lateral gapping, distraction of the fracture site and mal-reduction of the fractures [10, 12].

The Locking Compression Plate (LCP) distal ulna hook plate fixation was a novel alternative fixation method [13, 14] (Fig. 2). In addition, case series with LCP distal ulna hook plate fixation had shown improved clinical outcomes [14]. The purpose of this retrospective cohort study was to determine if LCP distal ulna hook plate fixation resulted in improved outcomes compared to the IMS fixation.

## Methods

### Patient eligibility

**Fig. 3:**
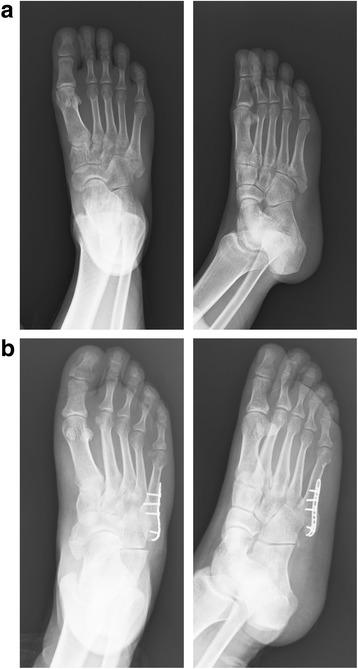
Pre- (**a**) and postoperative (**b**) radiographs of a fifth metatarsal base fracture that was treated surgically using the Locking Compression Plate (LCP) distal ulna hook plate fixation

**Fig. 4:**
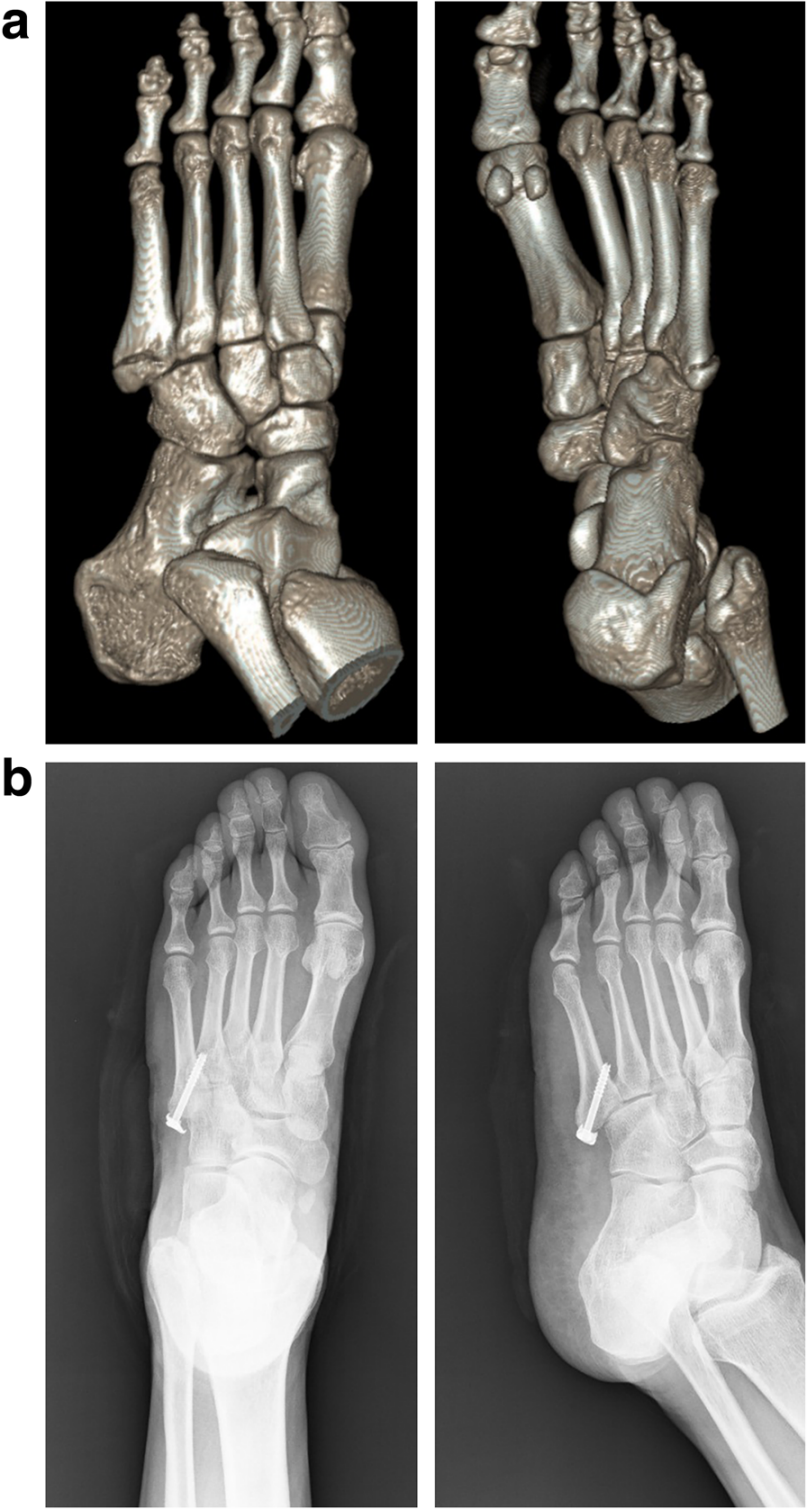
Preoperative 3D reformations of CT scans (**a**) and postoperative (**b**) plain radiographs of a fifth metatarsal base fracture that was treated surgically using the Intramedullary Screw

From July 2013 to July 2016, 42 patients (43 cases) with displaced avulsion Fifth Metatarsal Base Fractures were treated surgically and evaluated retrospectively. Ethical approval and informed consent from every single patient was obtained. Eligible patients were included in our study when they met the following criteria: (1) diagnosed with displaced (more than 2 mm) avulsion FMBFs; (2) over 18 years of age and in full possession of their mental faculties; (3) 4 days or less after injury; and (4) be treated with LCP distal ulna hook plate fixation (Fig. 3) or IMS (Fig. 4) and be follow-up until 12 months.

Patients with the following condition were excluded: (1) soft tissue injury: open fracture type Gustilo-anderson Type II or higher; (2) pathological fracture or re-fracture; (3) additional zone 2 and 3 fractures; and (4) bilateral fracture.

### Surgical treatment and rehabilitation protocol

All patients were surgically treated by a single, senior surgeon (ZHF) with the goals of anatomic reduction of the articular surface, achieve stable fixation and rebuilt the attachment of peroneus longus and brevis tendons. A lateral approach was used for direct visualization of the fractures as previously described. Patients in both cohorts were subject to the same postoperative rehabilitation protocol, which included no weight-bearing with short leg cast for 3 weeks. Radiographs were taken only on initial presentation to the clinic and in those patients with a great deal of pain clinically at the fracture site at the 6-week stage. Fracture union was defined radio graphically by bridging bone on at least 3 of 4 cortices.

### Clinical and function outcome assessments

The baseline characteristics including age, male, and smoking were collected. Patient-reported weight-beating time, pain relief, and clinical functional outcomes were prospectively collected at predetermined intervals of 3,6,9, and 12 months postoperatively. Subjective clinical outcomes were measured using the American Orthopedic Foot and Ankle Society Score (AOFAS). It was one of the most widely used clinician-reporting tools for foot and ankle conditions. Developed in 1994, AOFAS is a clinician-based score that measures outcomes on four different anatomic regions of the foot: The ankle-hindfoot, midfoot, metatarsophalangeal (MTP)-interphalangeal (IP) for the hallux, and MTP-IP for the lesser toes. Complications were also collected in our studies until the 12 months’ follow-up.

### Statistics

Statistical analyses were performed using STATA, version 10.0 (Stata Corporation, College Station, Texas, USA). We summarized continuous data with means and standard deviations (SDs). The two groups were compared with regards to continuous and categorical outcomes using the non-parametric T-test and Chi-square test respectively. We considered the conventional level of statistical significance as *p* < 0.05.

## Results

18 patients treated with IMS and 25 patients treated with LCP distal ulna hook plate fixation were included in the study. All the raw data was in the Additional file 1. The two cohorts had similar baseline characteristics, including mean age (39.89 and 34.36 years, *p* = 0.4575), gender distribution (27.78% and 60.00% male, *p* = 0.5226), and smoking rates (38.89% and 48.00%, *p* = 0.5528) (Table 1). For both cohorts, the surgery time was significantly less in patients with LCP distal ulna hook plate fixation cohort (40.94 and 53.5 min, *p* < 0.001). Time for partial weight-bearing (3.67 and 5.36 weeks, p < 0.001), full weight-bearing (6.52 and 8.48 weeks, p < 0.001), and bony union (7.49 and 9.64 weeks, *p* = 0.0053) was significantly less in patients with LCP distal ulna hook plate fixation cohort compared to IMS cohort. There was no significant difference in the time for return to daily life (12 and 12.52 weeks, *p* = 0.4192), pain scores before surgery (7.72 and 8, *p* = 0.2432), pain scores at 3 (4.61 and 4.64, *p* = 0.9072), 6 (3.56 and 3.68, *p* = 0.4589), 9 (2.39 and 2.68, *p* = 0.0856), and 12 weeks (0.44 and 0.52, *p* = 0.6346) after surgery, AOFAS before surgery (43.61 and 44.24, *p* = 0.4554), AOFAS at 3 (74.17 and 74.16, *p* = 0.9931), 6 months (76.67 and 76.6, *p* = 0.7879) after surgery between the two cohorts. While, the AOFAS scores at 9 (82.06 and 78.64, *p* < 0.0001), 12 months (93.56 and 87.8, p < 0.0001) after surgery were significantly higher in patients with LCP distal ulna hook plate fixation cohort (Table 2). One patient immigrates to Canada, we missed the follow up. There was no significant difference in the complications after surgery between the two cohorts.

**Table 1:** The baseline characters of patients

	PF *n* = 18	IMS *n* = 25	*p*-Value	Test
Age	39.89 ± 2.739	34.36 ± 1.977	0.4575	Unpaired T test
Male	27.78% (5)	60.00% (10)	0.5226	Chi-square test
Smoking	38.89% (7)	48.00% (12)	0.5528	Chi-square test

*PF* plate fixation, *IMS* Intramedullary screw

**Table 2:** Surgical Results of patients with Fifth Metatarsal Base Fracture

	PF (n = 18)	IMS (n = 25)	P	Test
Time for surgery*	40.94 ± 1.18	53.52 ± 0.66	**<0.0001**	Unpaired T test
Time to partial weight-bearing*	3.67 ± 0.14	5.36 ± 0.13	**<0.0001**	Unpaired T test
Time to full weight-bearing*	6.52 ± 0.12	8.48 ± 0.19	**<0.0001**	Unpaired T test
Time for bony union*	7.49 ± 0.10	9.64 ± 0.61	0.0053	Unpaired T test
Time for return to daily life	12 ± 0.45	12.52 ± 0.42	0.4192	Unpaired T test
Pain before surgery	7.72 ± 0.21	8 ± 0.13	0.2432	Unpaired T test
Pain 3 weeks	4.61 ± 0.18	4.64 ± 0.16	0.9072	Unpaired T test
Pain 6 weeks	3.56 ± 0.15	3.68 ± 0.10	0.4589	Unpaired T test
Pain 9 weeks	2.39 ± 0.14	2.68 ± 0.10	0.0856	Unpaired T test
Pain 12 weeks	0.44 ± 0.12	0.52 ± 0.10	0.6346	Unpaired T test
AOFAS before surgery	43.61 ± 0.56	44.24 ± 0.57	0.4554	Unpaired T test
AOFAS 3 months	74.17 ± 0.63	74.16 ± 0.46	0.9931	Unpaired T test
AOFAS 6 months	76.67 ± 0.19	76.6 ± 0.15	0.7879	Unpaired T test
AOFAS 9 months*	82.06 ± 0.12	78.64 ± 0.11	**<0.0001**	Unpaired T test
AOFAS 12 months*	93.56 ± 0.25	87.8 ± 0.17	**<0.0001**	Unpaired T test
Complication delayed union	2	3	>0.9999	Fisher’s exact test
Complication nonunion	0	0	–	–
Complication infection	0	0	–	–

*PF* plate fixation, *IMS* Intramedullary screw, *AOFAS* American Orthopedic Foot and Ankle Society

*and bold means *P* < 0.05

## Discussion

The main finding of this study is the efficacity of the fixation using a LCP distal ulna hook plate for a novel approach. There were only 4 studies studied the plate in the treatment of FMBFs [13, 15–17]. Three studies had evaluated the results of surgical treatment of zones I and II FMBFs using a mini-hook plate [13, 15, 16]. The fourth investigated the biomechanical comparison of IMS versus Low-Profile Plate fixation of a jones fracture [17]. There has been no previous comparison study investigating pain and functional outcome following treatment with LCP distal ulna hook plate and IMS, and our study is the first comparative retrospective cohort study to compare LCP distal ulna hook plate and IMS in displace evulsion FMBFs. Our finding showed that LCP distal ulna hook plate fixation as an alternative fixation method was better therapy for the displaced avulsion FMBFs than IMS fixation. LCP distal ulna hook plate fixation had a short surgery time and improved functional performance.

FMBFs can be challenging because of its subcutaneous location [18]. IMS as a precuneus technical was widely used in clinic and accepted as the standard of surgical treatment of these fractures [12]. Meanwhile, the screw system was developed, allowing surgeons to choose among 4.5-, 5.5-, and 6.5-mm solid stainless steel screws. However, it was difficulty to fix the small avulsion fractures with screws and there were several complications associated with them, such as irritability of screws head, injury of peripheral nerve, bone nonunion because of small diameter, and secondary fractures because of large diameter [12]. Alternatively, LCP distal ulna hook plate may be a good choice. This plate has several advantages: (1) the fifth metatarsal tuberosity can be grasped tightly by the plate hook to maintain the stability of the peroneal tendons adhesion; (2) as a checkered plate, the re-displaced of fractures can be reduced. Joint surface collapse can be prevented by the support function of this plate; (3) this plate had good histocompatibility; (4) the fifth metatarsal’s bending curvature fitted to the LCP distal ulna hook plate; and (5) low profile, obtuse edge and polishing surface can reduce the irritability of the soft tissue [17]. Vorlat, Achtergael and Haentjens reported that the most significant predictor of a poor functional outcome after these injuries was a prolonged period of non-weight-bearing [19]. The advantage functional outcome of LCP distal ulna hook plate was related to the early weight-bearing.

The retrospective cohort design of this study has several strengths. All cases were performed by a single senior surgeon (ZHF) using the same surgical approach, and all functional outcome evaluations were completed by a single senior physical therapist (SJZ) for precision. In addition, complications in both groups minimal. Limitations of this study include only twelve months of outcomes postoperatively, the total number of patients were small, and the radiographic evaluation was incomplete. Nowadays, we are focus on the effectively of the fifth metatarsal fractures anatomy plate.

## Conclusions

This study suggests that the use of LCP distal ulna hook plate fixation improves patients’ outcomes postoperatively.

## Additional file

Additional file 1: The file contains the raw data of the age, gender, smoking, time for surgery, time for partial weight-bearing, time for full weight-bearing, time for bone union, return to daily life, pain before surgery, pain 3 weeks, pain 6 weeks, pain 6 weeks, pain 12 weeks, AOFAS before surgery, AOFAS 3 months, AOFAS 6 months, AOFAS 9 months, AOFAS 12 months, delayed union, nonunion, and infection. (XLSX 32 kb)

## Abbreviations

AOFAS: American Orthopedic Foot and Ankle Society
FMBFs: Fifth Metatarsal Base Fractures
IMS: Intramedullary screw
LCP: Locking Compression Plate
PF: Plate Fixation
